# Cancer initiating-cells are enriched in the CA9 positive fraction of primary cervix cancer xenografts

**DOI:** 10.18632/oncotarget.13625

**Published:** 2016-11-25

**Authors:** Delphine Tamara Marie-Egyptienne, Naz Chaudary, Tuula Kalliomäki, David William Hedley, Richard Peter Hill

**Affiliations:** ^1^ Ontario Cancer Institute/Princess Margaret Cancer Centre, University Health Network and Campbell Family Institute for Cancer Research, Toronto, Ontario, M5G2M9, Canada; ^2^ Department of Medical Oncology, Princess Margaret Cancer Centre, Toronto, Ontario, M5G2M9, Canada; ^3^ Department of Medical Biophysics, University of Toronto, Toronto, Ontario, Canada; ^4^ Department of Laboratory Medicine and Pathology, University of Toronto, Toronto, Ontario, Canada; ^5^ Department of Radiation Oncology, University of Toronto, Toronto, Ontario, Canada

**Keywords:** cervix cancer, xenogratfs, stem cells, hypoxia, carbonic anhydrase 9

## Abstract

Numerous studies have suggested that Cancer Initiating Cells (CIC) can be identified/enriched in cell populations obtained from solid tumors based on the expression of cell surface marker proteins. We used early passage primary cervix cancer xenografts to sort cells based on the expression of the intrinsic hypoxia marker Carbonic Anhydrase 9 (CA9) and tested their cancer initiation potential by limiting dilution assay. We demonstrated that CICs are significantly enriched in the CA9^+^ fraction in 5/6 models studied. Analyses of the expression of the stem cell markers Oct4, Notch1, Sca-1 & Bmi1 showed a trend toward an increase in the CA9^+^ populations, albeit not significant. We present evidence that enhanced autophagy does not play a role in the enhanced growth of the CA9^+^ cells. Our study suggests a direct *in vivo* functional link between hypoxic cells and CICs in primary cervix cancer xenografts.

## INTRODUCTION

The cancer stem cell model of carcinogenesis argues that only a subset of cells in a tumor, the cancer stem cells (CSC) also termed cancer-initiating cells (CIC), are capable of regenerating the heterogeneous population composing a tumor when transplanted into immune-deficient mice [1, 2]. Such CICs can be enriched in cell populations obtained from solid tumors of diverse origins, i.e. brain, breast, colon, pancreas, head and neck, based on the expression of cell surface marker proteins, the most commonly used being CD133 and CD44, sometimes in combination with other surface markers such as CD24 or CD166 [3–8].

It is well established that normal stem cells of various tissues reside in a niche or microenvironment which tightly influences their fate and self-renewal [9–11]. Hematopoietic stem cells reside in an hypoxic (low oxygen (O_2_) levels) niche that regulates their metabolic and quiescence states [12–14]. There is also accumulating evidence for a role of such a critical microenvironment for CSCs, particularly a role for hypoxia-inducible factors [15, 16]. Mostly *in vitro* studies also show that the hypoxia inducible factors HIF1α or HIF2α, may control the maintenance and self-renewal of glioma CSCs [17–19].

In tumors, the abnormal, poorly organised tumor vasculature leaves areas with insufficient blood supply, creating areas of hypoxia [20]. Hypoxia can be measured in tumors via the expression of intrinsic hypoxic markers [21, 22]. These markers include glucose transporter 1 (Glut 1) and Carbonic Anhydrase 9 (CA9) [22]. A recent review has emphasized the widespread expression of CA9 in human cancers and related it to both poor outcome and stem cell markers [23]. Hypoxia has been linked with the experimental increase of the expression of the surface marker CD133 [19, 24–28] and with the concomitant exhibition of stem-like characteristics such as decreased differentiation [24, 26, 27]. The expression of regulators of stem cell maintenance (Oct4) and stem cell pathways (Notch, Wnt) are reported to be modified under experimental hypoxia [26–30]. Colocalization of markers of hypoxia and CSCs were reported in glioblastoma and pancreas tumors [17, 31] and in neuroblastoma cell line-derived xenografts, a highly tumorigenic side population was demonstrated to migrate towards areas of hypoxia [32]. Furthermore a recent study has reported that the hypoxia-inducible factor CA9 can play a role in maintenance of the CSC phenotype [33].

We have recently established a unique library of patient-derived early primary cervix cancer xenografts transplanted orthotopically (hereafter termed OCICx) that exhibit a good correlation with the original patient samples for characteristics of the tumor microenvironment [34]. Furthermore we, (and others) have previously reported that CA9, an hypoxia surface marker, can be successfully used to sort hypoxic cervical carcinoma cells originating from cell line-derived xenografts [35, 36]. Here we tested the hypothesis that CICs reside in CA9 positive fractions of solid cervical cancers by establishing limiting dilution assays (LDAs) of differentially-sorted CA9 populations from the OCICx models and determining their subsequent CIC frequency (CIF). We demonstrate a direct *in vivo* functional link between the CA9 positive fractions in primary cervix cancer xenografts and their content of CICs.

## RESULTS

### Initial studies of CICs in unsorted populations from the xenografts

The OCICx models and ME180-derived xenografts were examined in parallel. Our initial work with CA9 sorting involved the ME180 cervix cancer cell line [35], and this allowed a direct comparison of the CIC proportion in the primary xenografts versus an established cell line. Initially we investigated the transplant site that would give the best opportunity to detect tumor growth from unsorted cell suspensions derived from the whole tumor. We tested three sites for injection of cells from the ME180-derived xenografts and two sites for cells from the OCICx models (Supplementary Table S1). For both types of xenografts, the intra-muscular site in NOD/SCID mice gave the highest take rate. Similar results were obtained using the more immunocompromised NSG mice. Tumors resulting from intra-muscular injections exhibit a good level of conservation of the original xenograft architecture (Supplementary Figure S1). We therefore subsequently used this site for all LDA experiments.

**Figure 1:**
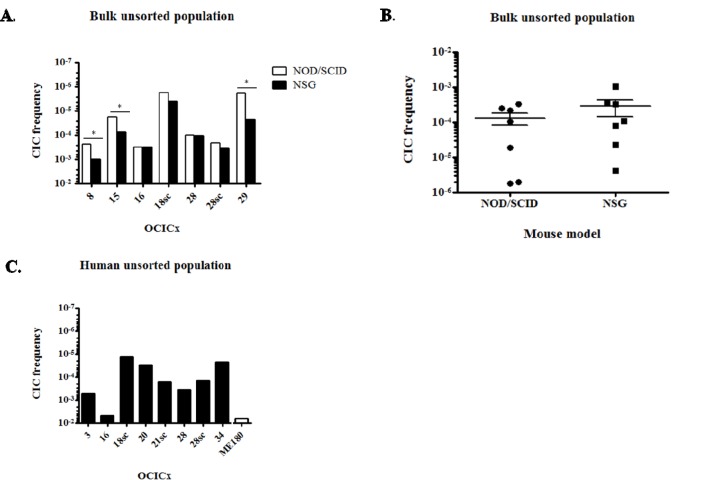
CICs are rare in OCICx models **A.** CIF of the bulk unsorted population of OCICx models that was determined by limiting dilution assay in NOD/SCID or NSG mice. This population represents the whole cell suspension without elimination of dead and mouse cells. The CIF is significantly different in NSG versus NOD/SCID mice for OCICx 8, 15 and 29 (*, p < 0.07). Data are from 1 LDA **B.** Comparison of the CIFs in the NOD/SCID and NSG mice for bulk unsorted populations of OCICx. Data are the CIF from (A). **C.** CIF of the human unsorted population from OCICx models tested in NOD/SCID mice. The CIF in the ME180-derived xenograft is shown for comparison.

Further LDA experiments on bulk unsorted cell suspensions for different orthotopic and subcutaneous models of OCICx and ME180-derived xenografts were performed in NOD/SCID and NSG mice simultaneously. The CIC frequency (CIF) varied from 1/3,000 for OCICx16 to 1/500,000 for OCICx18 and 29 in NOD/SCID mice (Table S2 and Figure 1A). Overall the CIF is low in the OCICx models with the median in the range 1-3 × 10^−4^ (Figure 1B). For OCICx28, for which a comparison was made between tumors growing in orthotopic and subcutaneous sites, the lower CIF for the subcutaneous site is not significantlydifferent from that for the orthotopic site (Table S2 and Figure 1A). Contrary to what was observed for the OCICx, the CIF is extremely high in the ME180-derived xenografts with a frequency of 1/50 (NSG) to 1/180 (NOD-SCID) (Table S2). The slightly higher value in the NSG mice was not significant but there was a significant increase in the CIF when NSG mice were used for the OCICx models 8, 15 and 29 (Figure 1A, p < 0.07). However, the median CIF for all the tests with the NSG mice is not significantly different from the median CIF for tests in NOD/SCID mice (Table S2, Figure 1B, p = 0.22).

Since we found that the percentage of human viable cells recovered from the different OCICx models varies greatly, we analysed the above data to estimate the CIF in the human cell fraction (Table 1 and Figure 1C), Correcting for these differences (Table 1, Figure 1C) brings the CIF in the unsorted human cell population to a minimum of 1/72,000 for OCICx18sc and a maximum of 1/200 for OCICx16, which is similar to the ME180 xenograft (1/180, Figure 1C). However, for all other primary cervix xenografts tested the CIF of the unsorted human population remains in the range observed for direct testing of the CIF of purified human populations from other tumor types (CIF < 1/2,000) [37, 38].

**Table 1:** CIC frequencies of the unsorted human cell population from OCICx models determined by limiting dilution assays in NOD/SCID mice

OCICx	3	16	18sc	20	21sc	28	28sc	34
**% human viable cells**	7.8 ± 1.1	8.6	9.4 ± 3.5	28 ± 2.5	22.9 ± 1.9	21.6 ± 2.1	32.5 ± 1.5	12.15 ± 3.6
**CIF human population**	1/1,800	1/200	1/72,000	1/32,000	1/6,000	1/2,800	1/7,000	1/43,000
**95 % confidence interval**	1/700-1/4,900	1/90-1/700	1/32,000-1/158,000	1/14,000-1/74,000	1/2,400-1/14,800	1/1,200-1/6,500	1/2,900-1/16,400	1/17,000-1/111,000

### CICs are enriched in the CA9^+^ fraction of the OCICx

**Figure 2:**
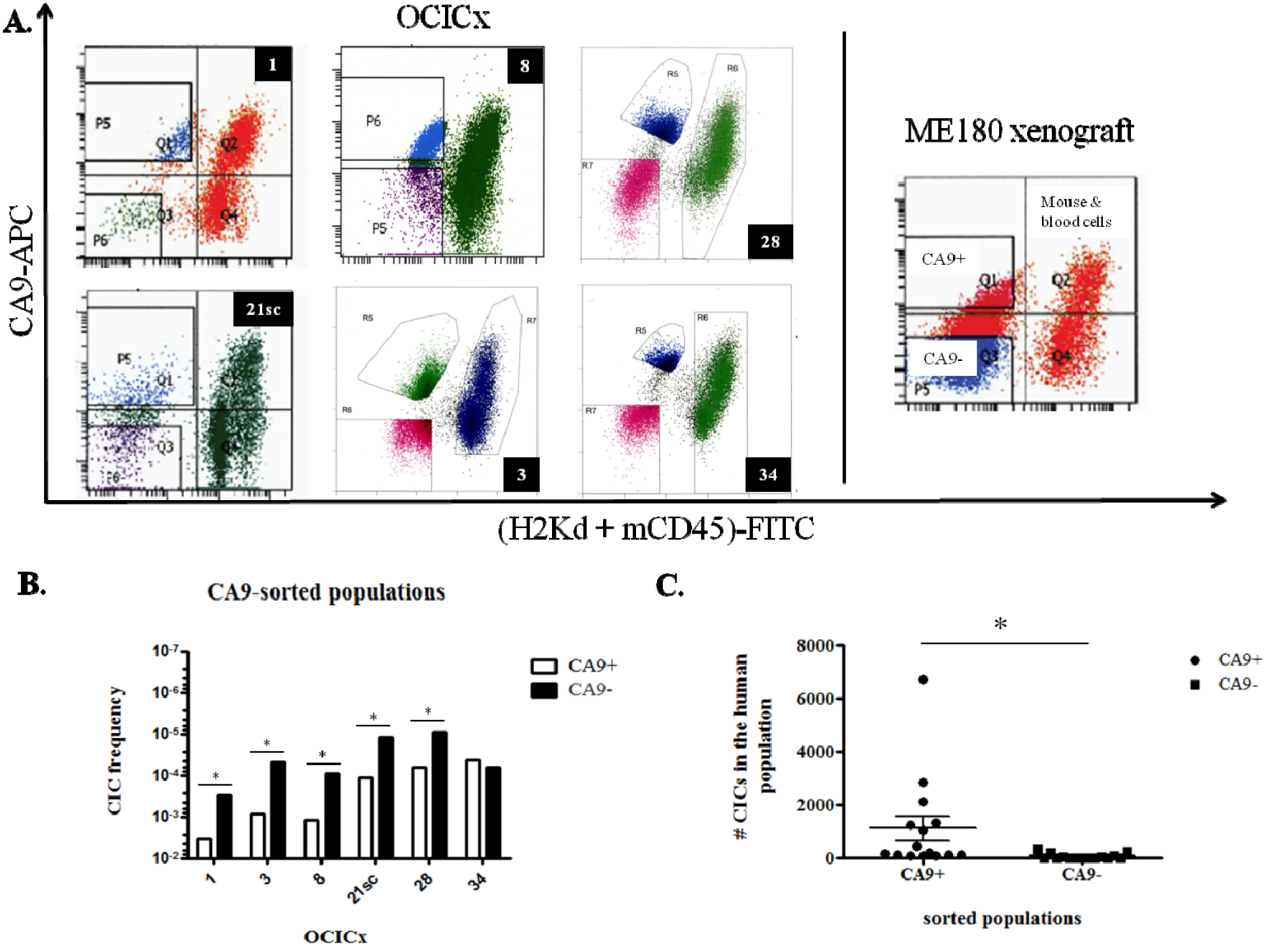
CICs are enriched in the CA9+ fraction of the OCICx **A.** Cells from the OCICx and ME180-derived xenografts were sorted based on the expression of CA9. Representative flow cytometry charts from OCICx (left panel) and ME180 xenografts (right panel). The CA9^+/−^ and mouse/blood cells populations are indicated in the ME180-derived xenograft panel. B-C. CICs are enriched in the CA9^+^ fraction of OCICx 1, 3, 8, 21sc and 28. **B.** CIF from pooled LDAs for CA9-sorted populations from OCICx models. Data is a combination of 3 independent experiments, except for OCICx34 for which n=4 (see Table 3). There is a significant increase in the frequency of CICs in the CA9^+^ population for OCICx 1, 3, 8, 21sc and 28 (*, p ≤ 0.0004). There is no statistical difference in the frequency of CICs between the CA9-sorted populations for OCICx34. **C.** Comparison of the number of CICs from the CA9-sorted fractions in the total human population. Data are from Table 3 for OCICx 1 to 28. The difference between the 2 populations is significant (*, p = 0.03).

Since we worked mainly with primary xenografts, we sorted viable human cells based on the exclusion of cells expressing the mouse histocompatibility complex class I H-2K[d] and mouse CD45, a marker of hematopoietic cells except erythrocytes (Figure 2A). Human cells are therefore (H-2K[d] + CD45)^−^ and the differentially expressing CA9 populations were sorted from this population. Table 2 summarizes the results of LDAs for the six OCICx models where we obtained regrowth of tumors from injected cells of both populations. The CIF of CA9^+^ populations from OCICx1, 3, 8, 21sc and 28 is significantly higher (approximately by a factor of 10) than the CIF of the CA9^−^ populations (Table 2 and Figure 2B). For OCICx 34, the difference between the two populations is not statistically different. In the ME180-derived xenograft, the CIF of both CA9-sorted populations are similar (Table 2), however, the CIF of both fractions appear somewhat higher than the CIF of the human unsorted population (see Figure 1C and Table S2). This may reflect the stress encountered by the cells during the sort procedure. For each experiment of OCICx 1, 3, 8, 21sc and 28 that contributed to the data in Table 2, the vast majority of CICs are found in the CA9^+^ fraction of the human population (see Table 3 and Figure 2C). Interestingly, the different experiments with the same model show some variability that might reflect differences between individual tumors (see Table S3). However, further detailed studies, beyond the scope of this study, would be required to determine if this is correct. The addition of (murine) stromal cells to the injected population for some of the models (3, 28,34) did not obviously impact the difference in the CA9^+^ and CA9^−^ fractions of the human tumor cells.

**Table 2:** CIC frequencies of sorted CA9^+/−^ populations from OCICx models and ME180-derived xenografts

OCICx	CIF CA9^+^ population	95% confidence interval	CIF CA9- population	95% confidence interval
**1**	1/200	1/140-1/600	1/3,000	1/1,050-1/9,800
**3**	**1/1,000**	**1/600-1/2,200**	**1/20,000**	**1/9,400-1/47,000**
**8**	1/800	1/490-1/1,300	1/11,000	1/4,300-1/28,000
**21sc**	1/8,000	1/3,800-1/20,000	1/80,000	1/27,000-1/260,000
**28**	**1/15,000**	**1/5,800-1/39,000**	**1/109,000**	**1/44,000-1/270,000**
**34**	**1/22,400**	**1/10,000-1/47,000**	**1/15,500**	**1/9,000-1/27,000**
**ME180**	1/700	1/200-1/2,500	1/600	1/200-1/1,800

**Table 3:** Number of CICs from the CA9^+/−^ in the total human viable population

OCICx	CIF of the CA9^+^ sorted population	Number of CICs from the CA9^+^ population in the total viable human cell population	CIF of the CA9^−^ sorted population	Number of CICs from the CA9^−^ population in the total viable human cell population
	1/140	**115**	1/2,050	**8**
**1**	1/460	**110**	1/3,700	**10**
	< 1/500	**> 75**	1/1,700	**20**
	1/750	**1330**	1/35,300	**30**
**3**	1/1,000	**2115**	1/7,000	**330**
	1/1,800	**460**	1/49,000	**20**
	1/60	**2830**	1/720	**240**
**8**	1/720	**150**	> 1/30,000	**< 4**
	1/8,100	**120**	> 1/50,000	**< 20**
	1/4,900	**70**	> 1/30,000	**< 10**
**21sc**	1/1,400	**6720**	> 1/50,000	**< 190**
	1/16,000	**200**	1/62,500	**50**
	> 1/6,000	**< 1050**	1/58,000	**100**
**28**	1/1,600	**1260**	1/91,000	**20**
	> 1/50,000	**< 75**	> 1/180,000	**< 20**
	> 1/25,000	**< 40**	> 1/25,000	**< 40**
**34**	1/19,600	**170**	1/11,500	**290**
	1/17,200	**440**	1/5,400	**1400**
	1/17,200	**60**	> 1/45,000	**< 20**

### CA9 is a marker of hypoxia in the OCICx

**Figure 3:**
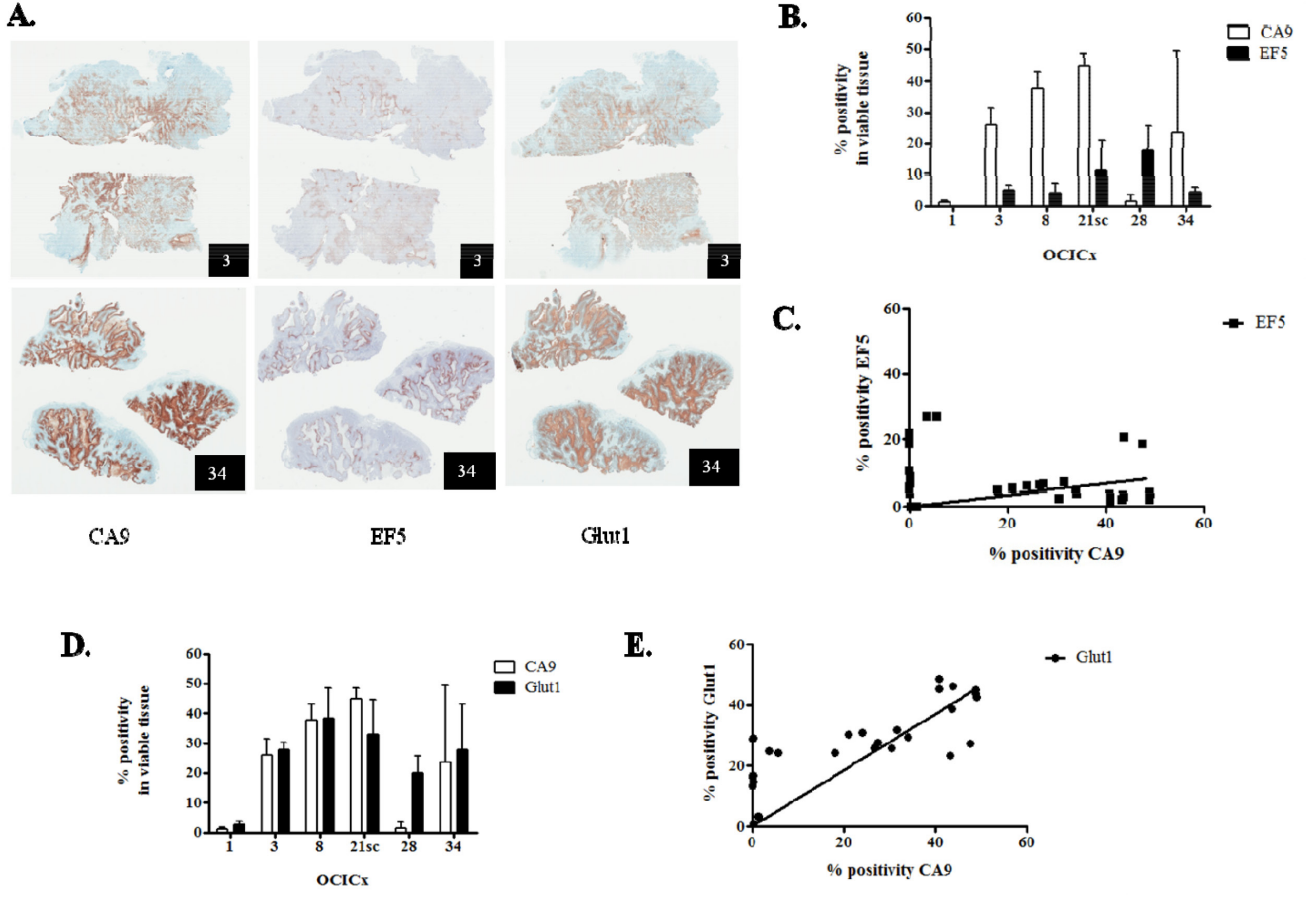
CA9 is a marker of hypoxia in the OCICx **A.** Immunostained serial sections of tumors from OCICx 3 and 34 (left, centre and right panels, CA9, EF5 and Glut1, respectively). The identity of the OCICx is indicated in the black box. **B-D.** Quantification of the immunohistochemical staining in viable tissue of CA9 and EF5 (B), and CA9 and Glut1 (D). Data are mean % positivity of the respective marker in viable tissue ± standard error from a minimum of 2 tumors per OCICx models with 1 slide per tumor (except for OCICx1, 8 and 28, see Table S3). **C-E.** Linear regressions of the quantification of CA9 and EF5 (C), and CA9 and Glut1 (E). Each point represents a tumor. The correlation between CA9 and Glut1 is highly statistically significant for the pooled data (spearman r = 0.75, p < 0.0001).

CA9 is a widely-used intrinsic hypoxia marker [22, 23, 33, 36, 39–42], and we and others have demonstrated its correlation with hypoxia in cervix cancer cell lines [35, 36], although it has recently been reported that CA9 expression does not correlate with pimonidazole staining in two cervix cancer xenograft models [43]. Accordingly, we analysed the relationship between hypoxia and CA9 in the OCICx models studied. We first investigated the extrinsic hypoxia marker EF5 and compared the extent of its binding to the expression levels of CA9 by immunohistochemistry. The staining for CA9 is more widespread than the staining of EF5 (Figure 3A, first and second columns). This translates into higher % positivity in viable tissue for CA9 compared to EF5 in all OCICx, except OCICx 28 (Figure 3B), and a weak but significant relationship between CA9 and EF5 staining (p = 0.02, Figure 3C). To further confirm that CA9 is a marker of hypoxia in the OCICx, we compared the expression levels of CA9 and Glut-1, another HIF-1α target gene recognized as an hypoxia marker by immunohistochemistry. There is a similarity in the staining patterns of CA9 and Glut1 (Figure 3A, first and third columns) and their expression levels are comparable, except again for OCICx28 (Figure 3D). Indeed, there is a strong linear relationship between CA9 and Glut1 staining (Figure 3E). Thus, CA9 exhibits a relationship with both EF5 and Glut1, and its expression strongly correlates (p < 0.0001) with Glut1, another intrinsic marker of hypoxia, consistent with the concept that CA9^+^ cells constitute an hypoxic fraction in the OCICx models, although it must be recognized that HIF-1α which drives expression of CA9 and GLUT-1 can be activated by other factors than hypoxia.

### CA9^+^ cells do not exhibit increase in the expression of stem cell markers in the OCICx models

**Figure 4:**
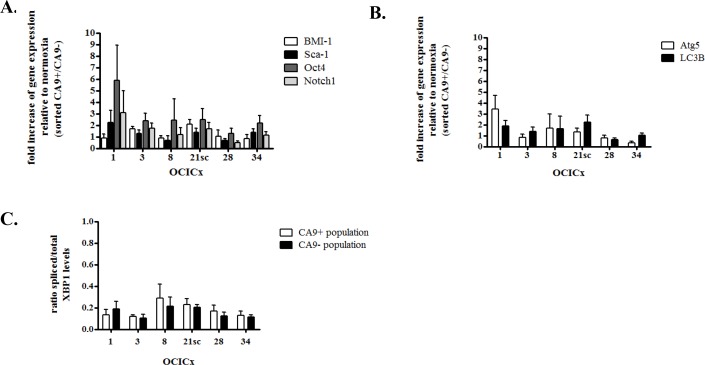
CA9+ cells do not exhibit increase in the expression of stem cell or autophagy markers in the OCICx 1, 3, 8, 21sc, 28 and 34 Gene expression changes in the sorted hypoxic (CA9^+^) and normoxic (CA9^−^) populations from the OCICx 1, 3, 8, 21sc, 28 and 34 determined by qRT-PCR. (A-B-C) Fold increase of gene expression related to normoxia (in CA9^+^ population relative to CA9^−^ population) for stem cell A. or autophagy/UPR markers B-C. Pooled data are shown as mean ± standard error. A minimum of 2 independent experiments were performed, depending on the OCICx model. There is no significant increase in the expression of any of the markers in the CA9^+^/CA9^−^ populations.

To determine whether the enrichment in CICs in the CA9 positive fraction translated into an increase in the expression of stem cell markers, we investigated the relative expression of the BMI-1, Sca-1, Oct4 and Notch1 markers in the CA9^+^/CA9^−^ populations from our five OCICx models by qRT-PCR. Although there is a trend for an increase in the expression of these markers in the CA9^+^ versus the CA9^−^ population, it is not significant (Figure 4A). The increase in the CA9^+^/CA9^−^ population rarely reaches over 2 fold for most genes. In OCICx1, Oct4 and Notch1 are expressed ≥ 2 fold in the CA9^+^ population compared to the CA9^−^ population, but not BMI-1 and Sca-1 for which the fold increase is similar to that observed for other OCICx models (Figure 4A). Thus, it does not appear that the enrichment in CICs in the CA9 positive fraction allows for detection of an increase in the expression of stem cell markers, possibly because of a low frequency of true CICs in these populations.

### CA9^+^ cells do not exhibit increase in the expression of autophagy/UPR markers in the OCICx models

Since the determination of CIF in an LDA relies on the ability of the injected populations to induce tumor growth, we investigated whether the increase in tumor take for the hypoxic CA9^+^ population might be due to the differential activation of survival mechanisms associated with hypoxia, namely upregulation of the unfolded protein response (UPR) and autophagy [44–46]. We tested the differential expression levels of markers of autophagy and UPR by qRT-PCR in the CA9^+^/CA9^−^ populations. The UPR contributes to hypoxia tolerance by transcriptional regulation of the autophagy genes LC3B and ATG5 [47]. XBP1, active under its spliced form, is a transcription factor implicated in both autophagy and UPR [44]. There was no significant increase in the expression of ATG5 and LC3B in the CA9^+^ versus CA9^−^ populations (Figure 4B). Similarly, no activation of XBP1 could be detected in the CA9^+^/CA9^−^ populations (Figure 4C). These findings strongly suggest that the increased tumor take-rate detected from CA9^+^ cells in the LDAs is unlikely to be due to enhanced survival of the cells post-injection because of autophagy and/or UPR.

## DISCUSSION

The limited availability of tissue from advanced cervical tumors led us to use early orthotopic cervix xenografts. Studies of CICs have often been performed with primary patient samples when large amounts of tissue are available, but the use of early-passage xenografts is quite common [4–6, 8, 48]. A better reproducibility in the experimental design has been argued to justify using early-passage xenografts [6, 48]. Nevertheless, it has demonstrated that passaging of xenografts can lead to changes in the histological architecture of the tumor and an increase in the frequencies of CICs [37, 38]. Therefore, we only used early-passage xenografts (passages 2-6). Furthermore, we demonstrated that CICs are rare in the OCICx, with a CIF of the estimated human population similar to that observed for the CIF of isolated human populations from primary tumor samples (CIF < 1/2,000, Table 2 and Figure 2) [37, 38]. In addition, we have previously demonstrated that the OCICx models exhibit a good correlation of markers of the tumor microenvironment with the patient biopsies [34], supporting the use of a marker of the tumor microenvironment to test the relationship between hypoxia and CICs in these models of cervix cancer. We also observed that tumours which grew from the sorted cell populations showed similar histology to the original tumour with heterogeneous levels of hypoxia in different tumours as expected (data not shown). Consequently, we believe it very likely that our findings, that CICs are enriched in the CA9 positive fraction of 5/6 of recently established primary cervix xenografts, reflect results that would be obtained directly from patient tumors.

Several laboratories have reported on the use of established cervical cancer cell lines, predominantly HeLa and SiHa to characterize cervical stem-like cells [49–52]. They used the *in vitro* surrogate assay of sphere formation in serum-free media, well-recognized for neural and breast CICs [17, 53] and assessed the self-renewal, migration and/or proliferation of the sphere-forming cells (SFC) and the effect of gene silencing, chemotherapeutic drugs or radiation on the behaviour of these cells [49–52]. While these studies constitute a first step towards the demonstration of CICs in the cervix, the use of long established cell lines limits their applicability due to the potential for *in vitro* adaptive changes in the cells. In fact our study showed that in the long established cervix cancer cell line ME180, the CIF is extremely high at 1/180 for the unfractionated population, which does not reflect what is generally observed for the OCICx models.

In one study, cervix cancer CICs from clinical samples 8/19 (42%) were reported to be capable of sphere formation, however, *in vivo* testing of the cancer-initiation capacity of the unfractionated sphere forming cells (SFC) was tested in only one small experiment [54]. These authors did report an increase in the expression of the surface marker CD44 for the SFC, but did not demonstrate its role in the isolation of CICs [54]. Other groups have reported an increase of the expression of CD44 or ALDH1 for cervix cancer SFC [51, 52]. We tested CD44 and CD24 as putative markers of CICs in two of our OCICx models and did not find a consistent relationship (see Table S4). Our robust *in vivo* quantitative assay to enrich for CICs could be used to test other cell surface and progenitor markers such as ALDH1, in combination or not with CA9, to potentially identify and further purify a subpopulation of cervix CICs from tumor samples or primary xenografts [50]. Further experiments are required to specifically isolate a CIC population from the OCICx models and allow a thorough examination of putative stem cell characteristics. Even though the CICs in the CA9+ (hypoxic) populations were enriched this was insufficient to allow us to characterize putative CSCs by determining the expression levels of stem cell markers by qRT-PCR in the sorted populations. Unfortunately low levels of cell recovery and RNA content from the sorted cells used for RNA extraction (except for OCICx1) limited these studies and their conclusions.

Previous reports have shown that hypoxia is critical for CICs in various different cancer types [17–19, 32, 33, 55, 56]. A few studies have also shown colocalization of markers of CICs and of hypoxia in tumors [17, 31, 32], or a direct influence of native tumor hypoxia on the behaviour of CICs [32]. Here, we report a direct functional link between CICs and the hypoxia marker CA9 by sorting CA9 positive cells and demonstrating that CICs are enriched in this fraction in five out of the six OCICx that were tested. For these five models, the number of CICs in the total human population is at least three times and up to fifty times higher in the CA9^+^ fraction. Furthermore, for all the models, the cancer-initiating potential appears to reside primarily in the CA9 positive fraction. Our data are consistent with a recent publication where hypoxia created by anti-angiogenic agents was reported to be responsible for an increased proportion of breast CSCs [57].

CA9 has been reported as a poor prognostic marker in cervix cancer, although a number of studies have found evidence for a correlation [40, 58–60]. A review of various endogenous hypoxia markers in cervix cancer including CA9 argued that they had limited prognostic value relative to direct measurements of hypoxic levels [61]. These results may reflect the fact that HIF-1α can be upregulated by a variety of factors and/or that hypoxia can play a variety of roles in the survival of tumour cells [62]. However, we and others have demonstrated that a sorted CA9^+^ population from ME180- or SiHa-derived xenografts constitute an hypoxic population [35, 36]. We confirmed that expression of CA9 showed a positive and highly significant correlation with another intrinsic marker and HIF-1α target, Glut1. There was also a correlation with the binding of the extrinsic hypoxia marker EF5 in serial sections but this was much weaker. Because of previous reports investigating the relationships between CA9 and extrinsic markers of hypoxia (the bioreductive drugs EF5 or pimonidazole), we did not expect CA9 and EF5 expression and binding, respectively, to correlate exactly. This is expected from the difference of O_2_ levels required to trigger the expression of CA9 and EF5 binding. For EF5 this binding is optimum at very low O_2_ levels (0.01-0.03% O_2_), whereas HIF-1α-dependent expression of CA9 is linked to 10-fold higher O_2_ levels (~ 0.1-1% O_2_) [63]. Depending on the tumor analysed, the level of colocalization between CA9 and pimonidazole in cervix tumors varies, with some levels of non-overlap even when the colocalization is excellent [36, 41, 42]. Despite a similarity in the pattern of expression/binding of CA9 and pimonidazole, the surface area of CA9 expression extends beyond the area of pimonidazole binding [36], resembling what we observed between CA9 and EF5. Glut1, on the other hand, is also a HIF-1α target gene induced under hypoxia and its expression has been shown to correlate significantly with CA9 in cervix tumors from patients [41, 64]. Indeed, we observe a highly significant correlation between the expressions of these intrinsic markers suggesting that they reflect HIF activity. Despite these observations it must be recognized that HIF-1α activity can be influenced by factors other than hypoxia, so this may also influence the correlation between CA9 or GLUT-1 expression and extrinsic markers of hypoxia such as EF5 or pimonidazole in certain tumours. A recent study has reported very limited concordance between pimonidazole staining and CA9 expression in two transplantable cervix cancer xenografts further emphasizing the heterogeneity in tumours [43].

CA9 and Glut1 have their own function in tumor cells that could influence the behaviour of the cells expressing them. CA9 is a member of the α-CA family of isozymes, the carbonic anhydrases [65]. These enzymes catalyse the reversible hydration of carbon dioxide to produce bicarbonate and a proton (H^+^). CA9 has been shown to play a role in the acidification of pH outside the cell (pH_e_) and to buffer the pH inside the cell (pH_i_) to allow biological reactions to take place [39, 65–67]. There is controversy about the link between hypoxia and the role of CA9 in pH modulation [68, 69], and whether low pH_e_ leads to an increase or decrease in CA9 expression, independent of hypoxia [69–72]. The induction of CA9 under hypoxia and its role in pH regulation are most probably linked and synergistic but we cannot rule out that pH regulation may have played a role in the behaviour of the CICs in the enriched CA9 positive fraction. Interestingly, pharmacological inhibition of CA9 catalytic activity leads to decreased overall tumor growth and reduction of the CICs population in a breast cancer cell line-derived xenograft model [73]. Although this report did not show that the loss of pH regulation by CA9 *per se* was necessary for this loss *in vivo*, it would be interesting to target CA9 catalytic activity in our OCICx and test whether an enrichment of CICs in the CA9^+^ fraction is still observed.

Overall, our results are consistent with a link between hypoxia and CICs of the cervix cancer xenografts. Hypoxia appears to provide a niche for the CICs, in which they can harbor CSC characteristics and drive tumor formation and growth, and eventually metastasis. However this microenvironment is not static, but evolves dynamically in relation with tumor growth, angiogenesis and fluctuations in blood flow that are well demonstrated to occur in tumors. Our results support the concept that CA9/hypoxia plays a role in the stem cell niche, and that their targeting in cervix patients with high levels of hypoxia could be beneficial to eradicate tumor growth and reduce recurrence.

## MATERIALS AND METHODS

### Cell culture and xenografts implantations

The human cervical carcinoma cell line ME180 was cultured as described previously [35]. The orthotopic cervical implantations were passaged orthotopically as described previously [34]. For subcutaneous (SBQ) xenografts, a piece of biopsy was implanted under the skin of (NOD)/SCID mice. The SBQ xenografts were further passaged subcutaneously. Details of the models used in the current study are shown in Table S5. All animals were bred in house at the Ontario Cancer Institute/Princess Margaret Cancer Centre (OCI/PMCC) small animal facility accredited by the Canadian Council on Animal Care. They were treated in accordance with approved animal care protocols at University Health Network (UHN).

### Single-cell suspensions and fluorescence-activated cell sorting (FACS) of cervix xenografts

The method was adapted from Chaudary & Hill [35]. All mice were injected intra-peritoneally with EF5 [2-(2-nitro1H-imidazol-1-yl)-*N*-(2,2,3,3,3-pentafluoropropyl) acetamide] up to twelve hours prior to sacrifice. A piece of extracted tumor was preserved in 10% neutral buffered formalin (VWR). Enzymatic dissociation of pooled OCICx or ME180 xenografts was performed with a solution of 1X Media 199 (Invitrogen) with a mix of Liberase Blendzymes 2 & 4 (Roche) or Liberase TH (Roche) and DNAse I (Sigma). The filtered cell suspension was rinsed several times with αMEM (PMH media facility) plus 2% FBS (Invitrogen). Viability was assessed using trypan blue exclusion. All subsequent procedures were performed at 4°C and in HBSS (Hank's balanced salt solution, PM media facility) without calcium and magnesium. The samples destined to be stained with CA9 were incubated with normal mouse serum (Invitrogen). Then antibodies specific for either the H2K-FITC (FITC mouse anti-mouse H-2K[d], 553565, BD Biosciences), CD45-FITC (FITC rat anti-mouse CD45, 553079, BD Biosciences), or CA9 (MAB2188, R&D Systems) protein were used in the appropriate samples for sorting. An anti-mouse antibody coupled with APC was used to detect CA9 (A21235, Invitrogen) for IHC studies. Prior to sorting, the cells were incubated with the viability dye Propidium Iodide (Biolegend) for viable cell discrimination. The cells were then sorted by FACS on a BD FACSAria II, or a BD MoFlow-XDP (BD Biosciences) at the Flow Cytometry Facility of the Toronto Hospital for Sick Children.

### Assay of cancer initiating potential: limiting dilution assay

For whole cell suspension, 3 or more doses of viable cells (determined by trypan blue staining) were injected into NOD/SCID or NSG mice. Doses of 10^3^ up to 10^6^ cells were used when possible, with 5 injections per dose. For CA9-sorted cells, when possible up to 3 groups of NOD/SCID mice (each group consisting of 5 doses) were injected with decreasing doses of cells for each population. For 3 of the models (OCICx 3, 28 and 34) sorted stromal cells (H2K + CD45)^+^ at a minimum of 10^5^ cells) were co-injected with the doses of CA9-sorted cells. Sorted and unsorted populations were mixed 1:1 with Matrigel (50ul final volume) prior to injection in hind leg muscles of mice. For sorted populations, all the mice received a sublethal irradiation dose (2Gy) with a γ-irradiator (Nordion) or with an X-ray irradiator (Precision X-ray) prior to the injections on the day of the implantation of the cells. For both types of experiment, tumor growth was monitored for up to 24 weeks.

### Real-Time quantitative PCR assay (qRT-PCR)

Total RNA was extracted from frozen cell pellets using the RNeasy Mini Extraction Kit (Qiagen) or the Total Purification Micro Kit (Norgen Biotek Corporation) from the CA9^+/−^ sorted cells according to the manufacturer's instructions. The qRT-PCR has been performed as described previously [35] except that 15 to 30 ng of RNA was used per reaction. The primer sequences are presented in Table S6 [74, 75]. The arithmetic mean of pooled data for L32, hsp90, ywaz and hprt1 were used to normalize the results.

### Immunohistochemical analysis

Immunohistochemical staining was performed by the Drug Development Laboratory at Ontario Cancer Institute as described previously [34]. Paraffin-embedded sections (4-μm thick) were dried in 60°C oven overnight. CA9 (Novus Biologicals, NB100-147, 1:2000) and Glut1 (Dako, A3536, 1:400) staining was carried out on a Ventana Benchmark XT fully automated slide preparation system, using the iVIEW 3,3′-Diaminobenzidine (DAB) detection kit. For EF5 (non-commercial ELK3.51 biotin conjugated mouse monoclonal antibody, 1:50 dilution, courtesy of Dr C. Koch) slides were dewaxed to water and heat-induced epitope retrieval was carried out in a T/T MEGA microwave histoprocessor and heated to 120°C in 10 mM Citrate buffer, pH 6.0. Sections were incubated overnight in primary antibody and detection was carried out with Streptavidin-horseradish peroxidase as secondary and DAB as substrate. The percentage of positivity of the immunohistochemical staining was quantified using the positive pixel counts program (% positivity = total positive area/total tissue area (pixels) in the viable tissue, Aperio Scanscope).

### Statistical analysis

Both the L-calc program (StemCell Technologies Inc, #28425 Version 1.1.1, October 2005) and/or the ELDA program (http://bioinf.wehi.edu.au/software/elda/) [76] were used to determine the frequencies of CICs and gave extremely similar results. The ELDA program was preferentially used. The significance between the median of the test of CIF between NOD/SCID and NSG mice and the number of CICs in the human population from the CA9-sorted populations were determined with a paired two-tailed Student t-test using the GraphPad software (GraphPad software Inc., San Diego, California). For the CA9^+/−^ populations, the differences between the CIF of sorted cells was determined using a one-way analysis of variance test included in the ELDA web tool. The linear regression and correlation analyses were generated using the GraphPad software. A two-tailed Spearman analysis was performed for all correlations. The significance between the fold change of expression of different markers between normoxic (CA9^−^) and hypoxic (CA9^+^) populations have been previously described [35].

## SUPPLEMENTARY FIGURE AND TABLES


