# Supplementary material for: Cancer initiating-cells are enriched in the CA9 positive fraction of primary cervix cancer xenografts
Source: Oncotarget. 2016 Nov 25;8(1):1392–404. doi: 10.18632/oncotarget.13625 (PMC5352063; doi:10.18632/oncotarget.13625)
Supplement: Supplementary file 1 [file oncotarget-08-1392-s001.pdf]

Cancer initiating-cells are enriched in the CA9 positive fraction of primary cervix cancer xenografts

SUPPLEMENTARY FIGURE AND TABLES

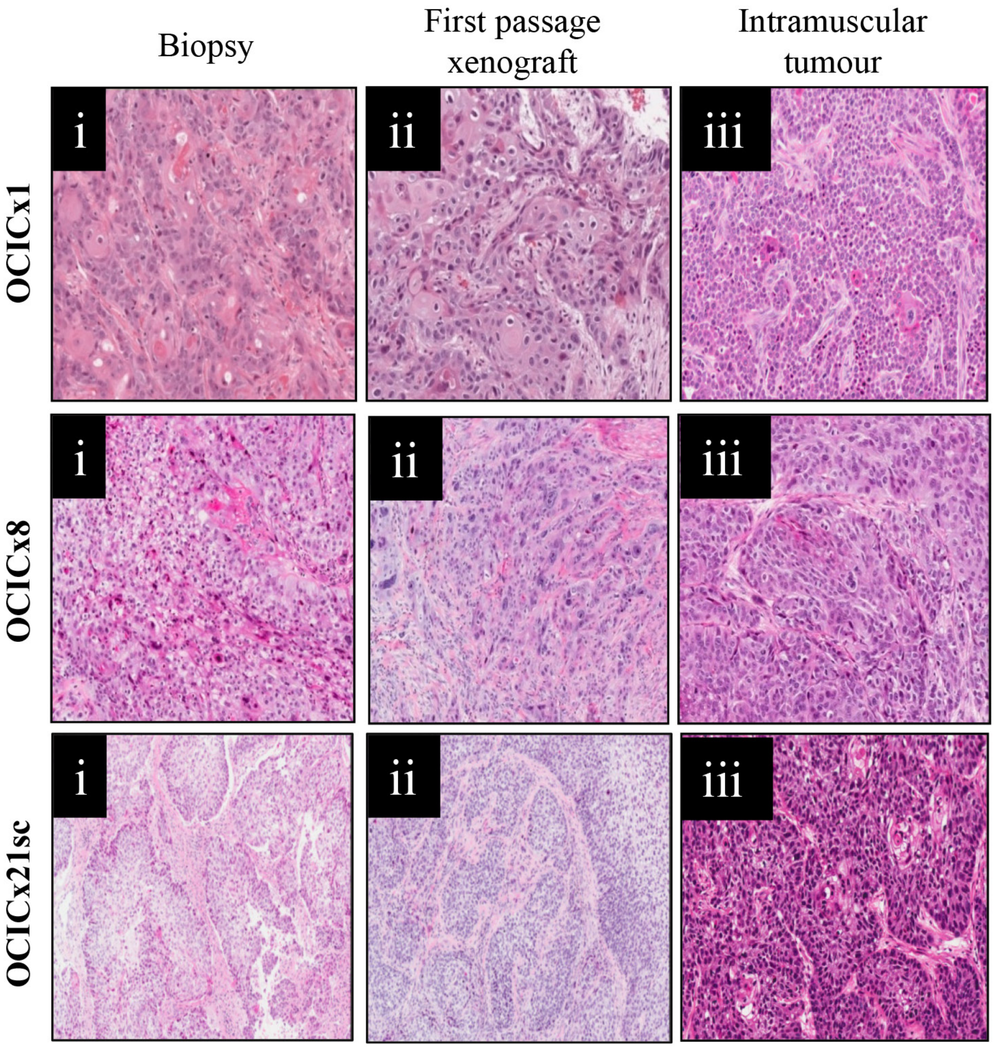

Supplementary Figure S1: OCICx primary cervix xenografts resemble the original patient tumors.

Supplementary Table S1: Tumor formation of ME180-derived and OCICx xenograft cells in different sites of injections in NOD/SCID and NSG mice, related to Figure 1

Xenografts	ME180		OCICx	
**Sites of injection**	**NSG**	**NOD/SCID**	**NSG**	**NOD/SCID**
**Intra-muscular**	10/10	10/10	5/5	14/14
**Subcutaneous**	7/8	9/10	5/5	8/9
**Sub-renal capsule**	6/8	0/10	-	-

10^5 cells were used for the injections, the number of injections is indicated. Data are from 1 experiment in the 2 mouse models for the ME180-derived xenografts. 2 experiments with each OCICx models 3 and 8 contributed to the reported OCICx values.

Supplementary Table S2: CIC frequencies of the bulk unsorted population from OCICx models and ME180-derived xenografts determined by parallel limiting dilution assays in NOD/SCID or NSG mice, related to Figure 1

OCICx	CIF in NSG mice	95% confidence interval	CIF in NOD/SCID mice	95% confidence interval
**1**	< 1/8,000	-	< 1/8,000	-
**8**	1/950	1/330-1/2,600	1/4,000	1/1,880-1/8,600
**15**	1/12,600	1/4,000-1/39,000	1/54,000	1/16,900-1/170,000
**16**	1/3,000	1/1,000-1/8,700	1/3,000	1/1,000-1/8,700
**18sc**	1/240,000	1/69,000-1/825,000	1/547,000	1/135,000-1/2,200,000
**28**	1/9,300	1/2,800-1/31,500	1/9,900	1/3,000-1/33,000
**28sc**	1/2,700	1/700-1/9,900	1/4,700	1/1,400-1/16,000
**29**	1/44,000	1/12,500-1/152,000	1/514,000	1/122,000-1/2,170,000
**ME180**	1/50	1/30-1/90	1/180	1/100-1/340

The CIF was determined from 1 LDA for each xenograft presented in each mouse model. The results are indicated as CIF with 95% confidence interval. sc, subcutaneous model, <1/8,000, estimated minimum frequency deduced from the maximum injected dose as the tumor take was 100%.

Supplementary Table S3: Number of CICs from the CA9$^{+/-}$ populations per 10^6 total cells, related to Figure 2 and Table 2

OCICx	Number of tumors that contributed to each experiment	CIF of the CA9$^+$ sorted population	Number of CICs in the CA9$^+$ population per 10^6 total cells	CIF of the CA9$^-$ sorted population	Number of CICs in the CA9$^-$ population per 10^6 total cells
	1	1/140	**35**	1/2,050	**1.5**
**1**	2	1/460	**5**	1/3,700	**2**
	2	< 1/500	**> 7**	1/1,700	**3**
	3	1/750	**5**	1/35,300	**0.2**
**3**	2	1/1,000	**4**	1/7,000	**0.08**
	2	1/1,800	**0.7**	1/49,000	**0.02**
	1	1/60	**15**	1/720	**5**
**8**	2	1/720	**3**	> 1/30,000	**< 0.02**
	2	1/8,100	**5**	> 1/50,000	**< 0.6**
	2	1/4,900	**0.1**	> 1/30,000	**< 0.2**
**21sc**	3	1/1,400	**0.2**	> 1/50,000	**< 0.3**
	3	1/16,000	**0.3**	1/62,500	**0.2**
	3	> 1/6,000	**< 0.05**	1/58,000	**0.15**
**28**	3	1/1,600	**0.45**	1/91,000	**0.15**
	1	> 1/50,000	**< 0.05**	> 1/180,000	**< 0.06**
	3	> 1/25,000	**< 0.04**	> 1/25,000	**< 0.04**
**34**	4	1/19,600	**0.09**	1/11,500	**0.13**
	3	1/17,200	**0.09**	1/5,400	**0.5**
	3	1/17,200	**0.12**	> 1/45,000	**< 0.16**

The number of CICs in the CA9-sorted populations was determined for each experiment that contributed to the data in Table 2. The number of CA9-sorted cells was normalised to the total number of sorted cells to determine the number of sorted cells per 10^6 cells. The subsequent number was then multiplied by the CIF of each sorted population to give the normalized number of CICs. The number of tumors used per experiment is indicated. < or > indicate that the maximum injected dose was used for CIF as no tumor was detected in the corresponding experiment.

Supplementary Table S4: CIC frequencies of sorted CD44$^{+/-}$ or CD24$^{+/-}$ populations from OCICx models, related to Figure 2

OCICx	CIF			
	CD44^{+} population (95% confidence interval)	CD44^{-} population (95% confidence interval)	CD24^{+} population (95% confidence interval)	CD24^{-} population (95% confidence interval)
1	**1/3,900** (1/1,500-1/10,000)	**1/1,200** (1/560-1/2,900)	-	-
3	**1/1,400** (1/560-1/3,400)	**1/5,900** (1/2,000-1/16,300)	**1/4,500** (1/1,500-1/13,400)	**1/4,200** (1/2,000-1/8,800)

The CIF was determined from 1 LDA for OCICx1 and from the combination of 2 independent LDAs for OCICx3. The results are indicated as CIF with 95% confidence interval. (-), no LDA was performed with CD24-sorted populations for OCICx1.

Supplementary Table S5: OCICx models

OCICx	FIGO staging	Tumor Type	Passage Number
**1**	IIB	SCC	3-4
**3**	IIA	SCC	4-5
**8**	IIB	AD	2-6
**15**	IIA	SCC	2-6
**16**	IIIB	SCC	4-6
**18**	IIB	SCC	3-4
**20**	IIIB	SCC	4-6
**21**	IIIB	SCC	2-5
**28**	IIB	SCC	2-5
**29**	IB	SCC	2-3
**34**	IB2	SCC	2-3

Punch biopsies from patients of the Princess Margaret Cancer Centre were sutured onto the cervix or implanted subcutaneously in (NOD)/SCID mice. The clinical status of the patient at the time of biopsy is indicated by the FIGO staging. SCC, squamous cell carcinoma, AD, adenocarcinoma. Only the tumors that were used in this work are presented. The range of passages at which the OCICx were used in this study is indicated.

Supplementary Table S6: Sequences of the primers used in the qRT-PCR experiments

Gene	Forward primer sequence (5’- 3’)	Reverse primer sequence (5’- 3’)
**ATG5**	GCAAGCCAGACAGGAAAAAG	GACCTTCAGTGGTCCGGTAA
**MAP1LC3B**	AACGGGCTGTGTGAGAAAAC	AGTGAGGACTTTGGGTGTGG
**XBP1 total**	GGCATCCTGGCTTGCCTCCA	GCCCCCTCAGCAGGTGTTCC
**XBP1 spliced**	CGCTTGGGGATGGATGCCCTG	CCTGCACCTGCTGCGGACT
**OCT4**	GACAACAATGAAAATCTTCAGGAGA	TTCTGGCGCCGGTTACAGAACCA
**BMI-1**	ATGTGTGTCCTTTG	AGTGGTCTGGTCTT
**SCA 1**	ATGGACTTCTCACACTACAAAG	TCAGAGCAAGGTCTGCAGGAGGACTC
**NOTCH1**	TCTGGACAAGATTGATGGCTACG	CGTTGACAACAAGGGTTGGACTC
**L32**	CAGGAGAGACACCGTCTGAACA	GAACCAGGATGGTCGCTTTCT
**YWAZ**	AATGCTTCACAAGCAGAGAG	TGCTTGTTGTGACTGATCGAC
**HSP90**	GTCTTCTGCTGGAGGTTCCTT	CTTTGACCCGCCTCTCTTCTA
**HPRT1**	CTAGTTCTGTGGCCACTCTGCT	GCCCAAAGGGAACTGATAGTC
